# Insights into Microbial and Metabolite Profiles in Traditional Northern Thai Fermented Soybean (Tuanao) Fermentation Through Metagenomics and Metabolomics

**DOI:** 10.3390/foods14173070

**Published:** 2025-08-30

**Authors:** Sivamoke Dissook, Patcharawadee Thongkumkoon, Pitiporn Noisagul, Chanenath Sriaporn, Sirikunlaya Suwannapat, Weeraya Pramoonchakko, Manida Suksawat, Thanaporn Kulthawatsiri, Jutarop Phetcharaburanin, Teera Chewonarin, Jetsada Ruangsuriya

**Affiliations:** 1 Department of Biochemistry, Faculty of Medicine, Chiang Mai University, Chiang Mai 50200, Thailand; sivamoke.dis@cmu.ac.th (S.D.);; 2 Center of Multidisciplinary Technology for Advanced Medicine (CMUTEAM), Faculty of Medicine, Chiang Mai University, Chiang Mai 50200, Thailand; 3 Office of Research Administration and Research Group on Space Weather and Cosmic Rays from Ground-Based Observations and Effects on Earth-Space Ecology, Chiang Mai University, Chiang Mai 50200, Thailand; chanenath.s@cmu.ac.th; 4 Department of Chemistry and Center for Innovation in Chemistry, Faculty of Science, Khon Kaen University, Khon Kaen, 40002 Thailand; sirikunlaya.s@kkumail.com (S.S.);; 5 Office of the President, Khon Kaen University, Khon Kaen University National Phenome Institute, Khon Kaen 40000, Thailand; 6 International College, Khon Kaen University, Khon Kaen 40000, Thailand; 7 Department of Systems Biosciences and Computational Medicine, Faculty of Medicine, Khon Kaen University, Khon Kaen 40000, Thailand

**Keywords:** Tuanao fermentation, shotgun metagenomics, metabolomics

## Abstract

Tuanao, a traditional Northern Thai fermented soybean product, was profiled with an integrated multi-omics workflow to clarify how microbes and metabolites co-evolve during household fermentation. Soybeans were fermented spontaneously for three days; samples from four time points were analyzed by shotgun metagenomics alongside 1H-NMR and UHPLC-ESI-QTOF-MS/MS metabolomics. *Bacillus* spp. (phylum Bacilliota) quickly supplanted early Enterobacterales and dominated the mature microbiome. The rise of *Bacillus* coincided with genes for peptide and carbohydrate utilization and with the accumulation of acetate, free amino acids (glutamine, leucine, alanine, valine) and diverse oligopeptides, whereas citrate and glucose-1-phosphate were depleted. This *Bacillus*-linked metabolic shift indicates that Tuanao is a promising source of probiotics and bioactive compounds. Our study provides the first system-level view of Tuanao fermentation and offers molecular markers to guide starter-culture design and quality control.

## 1. Introduction

Fermented soy products, including natto, tempeh, cheonggukjang, and kinema, have garnered global attention because fermentation not only softens the cotyledon matrix and improves protein digestibility but also enriches the beans with γ-poly-glutamic acid, antihypertensive peptides, and cardioprotective isoflavones [1,2,3]. These nutritional gains arise from a tightly choreographed microbial succession in which *Bacillus*, lactic acid bacteria (LAB), and, at times, fermentative yeasts drive proteolysis, saccharification, and secondary-metabolite formation [4,5]. Beyond their culinary value, several *Bacillus subtilis* and LAB strains isolated from fermented soy have been proposed as probiotics capable of modulating gut immunity and lowering serum cholesterol, underscoring the public-health importance of characterizing these foods at a systems level [6].

Tuanao is an artisanal sticky fermented soybean widely consumed in the mountainous northern provinces of Chiang Mai, Chiang Rai, Mae Hong Son, Lamphun, and Lampang. While Tuanao shares the characteristic “stringiness” of Japanese natto, it differs markedly in starter practice, often spontaneous or back-slopped rather than inoculated with a defined natto culture in ambient temperature (28–34 °C versus ~40 °C in industrial natto), and in downstream culinary use, serving chiefly as a condiment or mixed with chili pastes rather than eaten plain [7]. Household-level variability in raw materials, inoculation heuristics, and aeration thus creates lot-specific textures, flavors, and presumed health benefits [8]; nonetheless, previous investigations have provided only partial snapshots, such as plate counts of *Bacillus* colonies or 16S rRNA amplicon surveys without paired metabolite data, leaving the functional linkage between microbial succession and metabolite output largely speculative [9].

Raw material selection adds a further layer of complexity. Soybean cultivar, seed size, and seed-coat chemistry shape water absorption, endogenous enzyme activities, and microbial attachment, thereby influencing fermentation kinetics and the final organoleptic profile [10]. Even when inoculated with the same *B. subtilis* strain, cultivar-driven differences in protein and oligosaccharide composition can yield divergent volatile flavor and free-amino-acid signatures, underscoring the need for a holistic multi-omics view that simultaneously captures community composition and small-molecule dynamics. Although integrative studies combining shotgun metagenomics with untargeted metabolomics have begun to unravel such microbe–metabolite linkages in complex foods like kimchi, sourdough, and surface-ripened cheeses [11], no comparable analysis has yet been undertaken for Tuanao, creating a critical knowledge gap in both regional gastronomy and fermented-food science.

However, Tuanao has been the subject of several microbiological and chemical profiling studies. Collectively, these works show that *Bacillus* species are the principal fermenters in Tuanao, with *B. subtilis* dominating fermentation from start to finish [12]. Early culture-dependent studies and 16S rRNA surveys found that Bacillus can constitute over 50–80% of the community, while only minor fractions of lactic acid bacteria or other genera appear in fresh, naturally fermented samples [13,14]. Consistent with an alkaline fermentation, Tuanao is rich in proteases and amino acids: fermentation by *B. subtilis* releases abundant free amino acids (e.g., glutamate, lysine), enhancing umami flavor and nutritional value [15]. Thua Nao’s potent aroma, often described as pungent or “rotten” has been traced to volatile metabolites produced by Bacillus, notably alkylpyrazines (imparting nutty, roasted aromas) and short-chain fatty acids and ammonia (responsible for sweaty, pungent odors) [16,17]. Biochemical analyses further show that Bacillus fermentation increases health-related compounds: fermented soybeans have higher levels of antioxidant phenolics and aglycone isoflavones than unfermented beans.

However, prior studies relied largely on culture-based identification, 16S amplicon sequencing, or targeted chemical assays. No previous work has employed shotgun metagenomics combined with untargeted metabolomics for Thua Nao. Our current study, therefore, builds on the literature by providing a holistic, high-resolution view of Thua Nao’s fermentation ecosystem, identifying all microorganisms and a broad spectrum of metabolites, which allows us to link community functional potential with chemical outcomes in a way not possible in earlier analyses.

To address this gap, we profiled the dynamic metabolome of Tuanao over three days of fermentation using complementary ^1^H NMR and untargeted LC-MS, while characterizing the concomitant microbiome at strain-level resolution via shotgun metagenomics; we then integrated these datasets through correlation, co-occurrence, and network analyses to identify microbial drivers of key metabolites. By mapping the temporal interplay between community structure and chemical output, the present study establishes, for the first time, reliable multi-omics biomarkers of Tuanao quality. These findings provide a scientific foundation for rational starter-culture design, evidence-based quality-control schemes that preserve regional character while enabling industrial scale-up, and future investigations into the gut-health and functional-food potential of this culturally significant Thai fermentation product.

## 2. Materials and Methods

### 2.1. Sample Preparation

Dry soybeans were purchased from a specific company distributor in Thailand. The company is certified under multiple quality and safety standards, including HACCP (HACCP08068/102), GMP (GMP04003/014), FDA Thailand, and Thailand Trust Mark, ensuring consistency and traceability of the soybean source.

In the production of Tuanao, dry soybeans (1 kg per batch) were first rinsed to remove surface contaminants and then soaked overnight in water to facilitate rehydration. After soaking, the beans were boiled for 180 min. After cooking, the hot soybeans were transferred into 50 mL centrifuge tubes, providing adequate space for expansion and gas release, and the lids were loosely closed to allow for semi-anaerobic conditions conducive to fermentation. The tubes were then incubated at 37 °C to promote the growth of natural fermentative microorganisms.

Fermentation was conducted using three independent batches, designated as Lot 5 (L5), Lot 6 (L6), and Lot 9 (L9), to assess batch-to-batch variability. For each lot, samples were collected at four fermentation stages: Day 0 (unfermented), Day 1, Day 2, and Day 3. All experiments were performed in biological triplicates (*n* = 3) at each time point and for each lot.

### 2.2. NMR Spectroscopy

For ^1^H NMR spectroscopic analysis in our study, individual replicates were accurately weighed and meticulously ground with a solvent mixture of methanol, water, and chloroform in the ratio of 5:2:2 (*v*/*v*/*v*). The quantity of the solvent mix was precisely adjusted in relation to the sample weight, with 1 mL of solvent mix used per 100 mg of sample. Following centrifugation at 1000 g and 4 °C for 15 min, the upper aqueous phase was carefully collected and subsequently dried using a vacuum concentrator at 40 °C. The resulting dry extracts were stored at a very low temperature of −80 °C until further analysis. For the re-solubilization of the dried extracts, a buffer mixture containing 100 mM sodium phosphate at pH 7.4, made with D_2_O and inclusive of 0.1 mM 3-trimethylsilylpropionic acid (TSP) as a reference for chemical shifts, was used. The sample solution was then sonicated and filtered before being placed in an NMR tube, which was then inserted into a 400 MHz NMR spectrometer (Bruker, Billerica, MA, USA) for metabolic profiling using a Carr–Purcell–Meiboom–Gill (CPMG) pulse sequence [RD–90°–(ꞇ–180°–ꞇ)n–acquisition] that was applied to samples at 310 K (2ꞇn = 76.8 ms) in order to improve the visualization of signals generated from low-molecular-weight metabolites. A total of 64 scans were recorded into 72k data points with a spectral width of 20 ppm. The NMR spectral data were processed using MATLAB (R2015a) to adjust peak alignment and normalization using probabilistic quotient normalization (pqn). Metabolite identification was primarily conducted using Statistical Total Correlation Spectroscopy (STOCSY), a powerful correlation-based approach performed with in-house scripts in MATLAB R2020a. This method identifies signals from the same molecule by calculating the pairwise correlation of signal intensities across all sample spectra. A driver peak from a known resonance was selected, and all signals with a high correlation coefficient (r^2^ > 0.9) were assigned to the same molecule. The complete spectral fingerprint obtained from STOCSY for each metabolite was then rigorously confirmed by comparing chemical shifts (δ), J-coupling constants, and signal multiplicities against our in-house spectral database, the Chenomx NMR Suite, and the Human Metabolome Database (HMDB). The quantification of each metabolite was calculated according to the maximum intensity of the signal of interest compared to the maximum intensity and concentration of TSP. Data preprocessing, including binning, alignment, and probabilistic quotient normalization, was performed using the IMPaCTS toolbox (https://doi.org/10.5281/zenodo.3077413, accessed on 27 August 2025). The list of metabolites with their corresponding driver peaks used in STOCSY analyses is provided in Table S1, and representative STOCSY plots are presented in the Supplementary Information.

### 2.3. UHPLC-ESI-QTOF-MS/MS Analysis

The metabolic profiling was conducted at the Khon Kaen University National Phenome Institute (KKUNPhI). The samples were analyzed on a reverse-phase liquid chromatography platform. The separation was performed using a UHPLC system (Bruker, Bremen, Germany). A Bruker intensity solo HPLC C18 2.1 × 100 mm, 2 μm column (Bruker, Bremen, Germany) was used. The column temperature was set at 40 °C, and the autosampler temperature was set at 4 °C. Mobile phase A was 100% water with 0.1% formic acid (FA), and mobile phase B was 100% acetonitrile with 0.1% FA. The flow rate was set at 0.35 mL/min, and the elution gradient was set as follows: 99% A (0.0–2.0 min, 0.25 mL/min), 1% A (2.0–20.0 min, 0.25 mL/min), 99% A (20.1–28.3 min, 0.35 mL/min), 99% A (28.5–30.0 min, 0.25 mL/min). The injection volume of the sample was 2 μL applied for both positive and negative ionization polarity modes. The mass spectrometry was performed using a compact ESI-Q-TOF system (Bruker, Germany). Sodium formate solution (2 mM sodium hydroxide, 0.1% FA, 50% isopropanol) was directly injected as an external calibrant with a flow rate of 0.5 μL/min. The conditions in positive ionization polarity mode were as follows: mass range 50–1500 *m*/*z*, cone voltage 35 V, capillary voltage 4000 V, source temperature 200 °C, desolvation temperature 200 °C, desolvation gas flow of 8 L/min. The conditions in negative ionization polarity mode were as follows: *m*/*z* range: 50–1500 *m*/*z*, cone voltage 31 V, capillary voltage 4500 V, source temperature 200 °C, desolvation temperature 200 °C, desolvation gas flow of 8 L/min.

### 2.4. Metagenomics and Functional Annotation

Bacterial genomic DNA was extracted from 200 mg of fermented soybean collected on Days 0, 1, 2, and 3 from a total of three batches using the DNeasy^®^ mericon Food Kit (QIAGEN, Hilden, Germany, Cat. No. 69514), according to the manufacturer’s instructions. Genomic DNA was quantified and qualified using a Nanodrop spectrophotometer (Thermo Fisher Scientific, Waltham, MA, USA). The quantity and quality of the genomic DNA were assessed using a NanoDrop ONE (Thermo Fisher Scientific, USA). Library preparation was performed by fragmenting the DNA, which was then sequenced on the NovaSeq 6000 platform (Illumina, San Diego, CA, USA) paired-end 150 bp, with a total data output of 12 Gb (Novogene, Singapore).

Raw metagenomic sequences were processed through a comprehensive and fully automated analysis pipeline known as SqueezeMeta version 1.6.5 [18]. The initial phase of processing involved quality filtering and trimming using Trimmomatic, adhering to a minimum quality score threshold of 30 and retaining reads no shorter than 50 bp in length [19]. Subsequently, the quality-filtered reads from all samples were pooled and subjected to genome assembly using MetaSPAdes version 3.15.3 in coassembly mode, with specific k-mer parameters (-k 21, 33, 55, 77, 99, 127) being used to optimize assembly outcomes [20].

For the mapping of reads back to the coassembled contigs, Bowtie version 2.5.4 was used to align each sample’s reads individually, enabling the determination of sample-specific coverage profiles essential for the binning process [21]. The binning of contigs into metagenome-assembled genomes (MAGs) was performed using MetaBAT2version 2.18 [22], MaxBin version 2.2.7 [23], and CONCOCT version 1.1.0 [24], which leverage differential coverage information across the pooled samples. The resulting MAGs were compared, and representatives were selected using DAS Tool version 1.1.6. Dereplication of MAGs across the coassembled dataset was carried out using dRep version 3.4.2 with a 99% similarity threshold to yield a non-redundant set of representative bins.

Gene prediction within the prokaryotic communities was conducted using Prodigal, which facilitated the prediction of coding DNA sequences (CDSs) [25]. These sequences were then annotated against two major databases, namely eggNOG [26] and KEGG [27], by employing Diamond version 2.1.13, with an e-value cutoff of 0.001 for stringent annotation criteria [28]. Prokaryotic taxonomic annotation was achieved by conducting Diamond searches against the GenBank nr database, ensuring a comprehensive taxonomic profiling.

For the visualization of genome and functional abundance, R version 4.3.1 was employed alongside the ggplot2 package version 3.5.2 and SQMtools package version 1.7.2, enabling detailed graphical representation and interpretation of the data.

### 2.5. Statistical Analysis

Statistical analyses were performed using R version 4.3.1. Metagenomic data were processed using SQMtools version 1.7.2 and phyloseq [29] packages version 1.42.0 to handle taxa abundance and functional annotation data from KEGG databases. Metabolomic data from Nuclear Magnetic Resonance (NMR) spectroscopy and Ultra-High-Performance Liquid Chromatography–Electrospray Ionization–Quadrupole Time-of-Flight Mass Spectrometry (UHPLC-ESI-QTOF-MS/MS) were analyzed using principal component analysis (PCA) and partial least squares discriminant analysis (PLS-DA). PCA was performed with the R packages ‘FactoMineR’ [30] version 2.6, ‘factoextra’ version 1.0.7, and ‘ggfortify’ [31] version 0.5.16 to identify variations in metabolite compositions. The R package ‘mixOmics’ [32] version 6.22.0 was utilized for conducting PLS-DA and calculating variable importance in projection (VIP) scores to identify key metabolites.

Spearman’s rank correlation analysis was performed using ‘Hmisc’ version 5.1-0 and ‘corrplot’ [33] packages version 0.92 to explore relationships between microbial communities, metabolites, and KEGG pathways, with significance indicated at *p* < 0.05, *p* < 0.01, and *p* < 0.001. Heatmap visualizations were generated using the ‘ComplexHeatmap’ package version 3.15.

Alpha diversity indices, including Shannon and Simpson indices, were computed with the ‘phyloseq’ and ‘vegan’ [34] packages to assess bacterial community diversity across different fermentation lots and time points. Visualizations of alpha diversity were created using ‘ggplot2’.

Statistical significance for all analyses was set at an alpha level of 0.05. Normality and homogeneity of variances were evaluated using the Shapiro–Wilk and Levene tests, respectively, to guide the choice of appropriate statistical methods. Graphical representations were generated in R using relevant plotting libraries such as ‘ggplot2’version 3.5.2, ‘patchwork’ version 1.1.2, and ‘pheatmap’ version 1.0.12.

## 3. Results

### 3.1. Metabolite Profiling of Fermented Soybeans

#### 3.1.1. NMR Analysis

**Figure 1:**
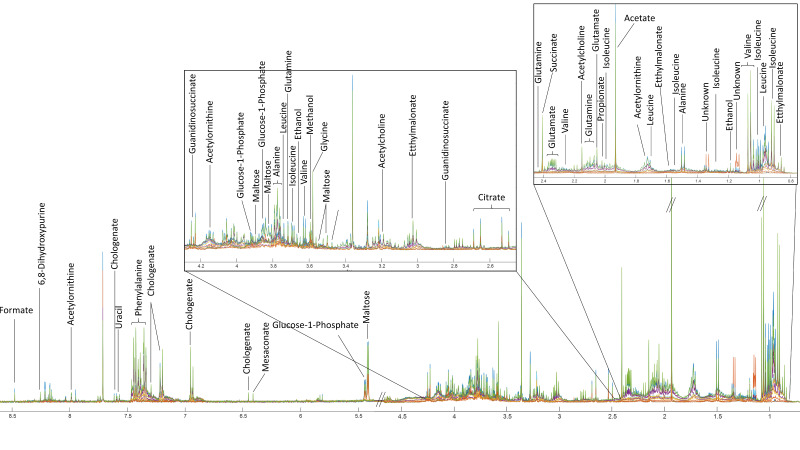
^1^H NMR CPMG spectra showcasing the metabolic profile of fermented soybeans. Each spectrum represents a different stage in the fermentation process with overlapping signals from various sugars and amino acids. Key resonances are indicated for metabolites including formate, phenylalanine, citrate, succinate, and unidentified compounds, among others. The figure was plotted using MATLAB R2021a version 9.10 (MathWorks, Inc., Natick, MA, USA).

**Figure 2:**
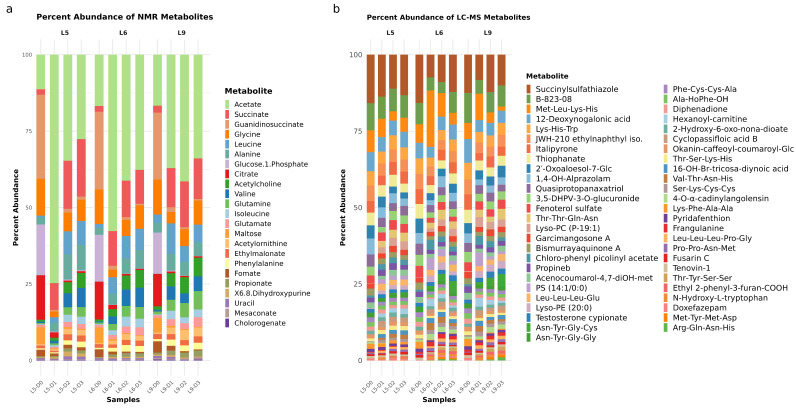
Relative abundance of Tuanao metabolites as detected from (**a**) NMR and (**b**) LC-MS.

Nuclear Magnetic Resonance (NMR) spectroscopy has been employed to elucidate the complex metabolic profile of fermented soybean samples. A ^1^H NMR spectrum highlighting key metabolite signals at different fermentation stages is shown in Figure 1. To complement the spectral data, the relative abundance of key Tuanao metabolites detected via ^1^H NMR is summarized in Figure 2a, highlighting distinct shifts in metabolite concentrations over the fermentation period. The NMR analysis has allowed for the identification of metabolites, ranging from amino acids to organic acids, sugars, and other significant bioactive compounds. See Table S1 for full assignments and chemical shifts. Our results demonstrate a dynamic metabolic shift corresponding to the fermentation time, with distinct patterns emerging at specific intervals. Notably, principal metabolites, such as isoleucine, leucine, and valine, showcased a significant change in their concentration, echoing the metabolic adjustments occurring during the fermentation process. The precise data obtained through NMR has enabled the identification of key biomarkers of fermentation, contributing to our understanding of soybean metabolism. These findings offer valuable insights into the biochemical pathways activated during soybean fermentation and lay a foundation for further investigation into optimizing fermentation conditions for enhanced nutritional outcomes.

#### 3.1.2. LC-MS Analysis

In addition to NMR spectroscopy, Ultra-High-Performance Liquid Chromatography–Electrospray Ionization–Quadrupole Time-of-Flight Mass Spectrometry (UHPLC-ESI-QTOF-MS/MS) was employed to further delineate the complex metabolite landscape of Tuanao fermentation, shown in Figure 2b. This high-resolution technique enabled the detection and relative quantification of a wide array of metabolites, providing a comprehensive dataset shown in Table S2. The identified compounds spanned various chemical classes, including acylcarnitines, complex fatty acids, steroids, and numerous naturally occurring compounds and oligopeptides. The LC-MS profiles offered a complementary perspective to the NMR data, capturing a different and broader range of molecular species. These profiles revealed significant alterations in metabolite abundances throughout the various stages of fermentation. The detailed data obtained from this analysis were crucial for subsequent investigations to identify fermentation-specific metabolic signatures and to correlate these with the observed microbial community dynamics, which are further discussed in the section. This workflow lacked MS/MS fragmentation and used an ESI ion source suboptimal for low-abundance nonpolar compounds; therefore, annotated masses were interpreted with caution, and improbable fat-soluble vitamin hits were removed.

### 3.2. Metagenomic Analysis

#### 3.2.1. Tuanao Communities Were Mainly Associated with Bacilliota and Enterobacterales

**Figure 3:**
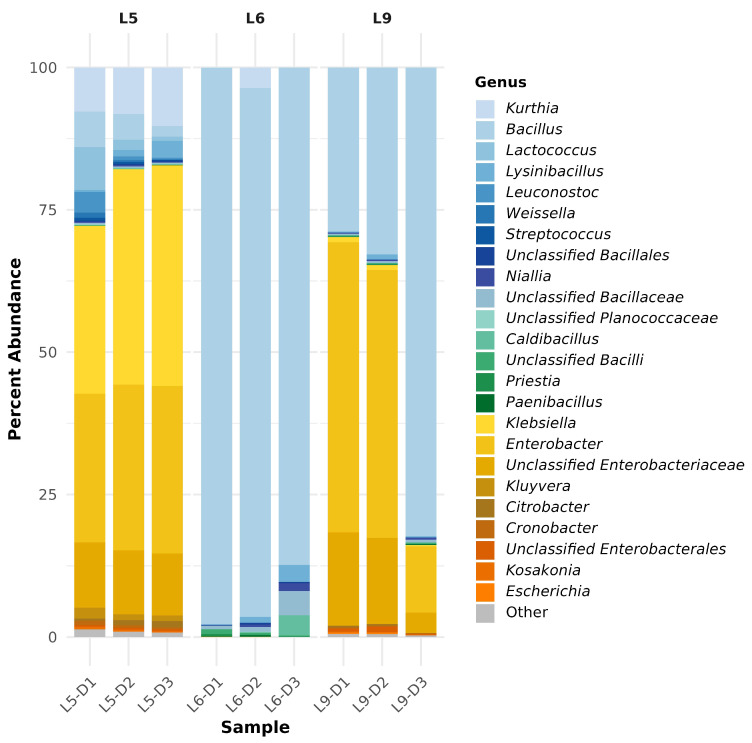
Relative abundance of detected bacterial genera across different fermentation stages of Tuanao from L5-D1 to L9-D3: Only those with >0.1% relative abundance were included. Legends with blue and green shades are of Bacilliota lineages, and legends with yellow and red shades are of *Enterobacterales* lineages.

**Figure 4:**
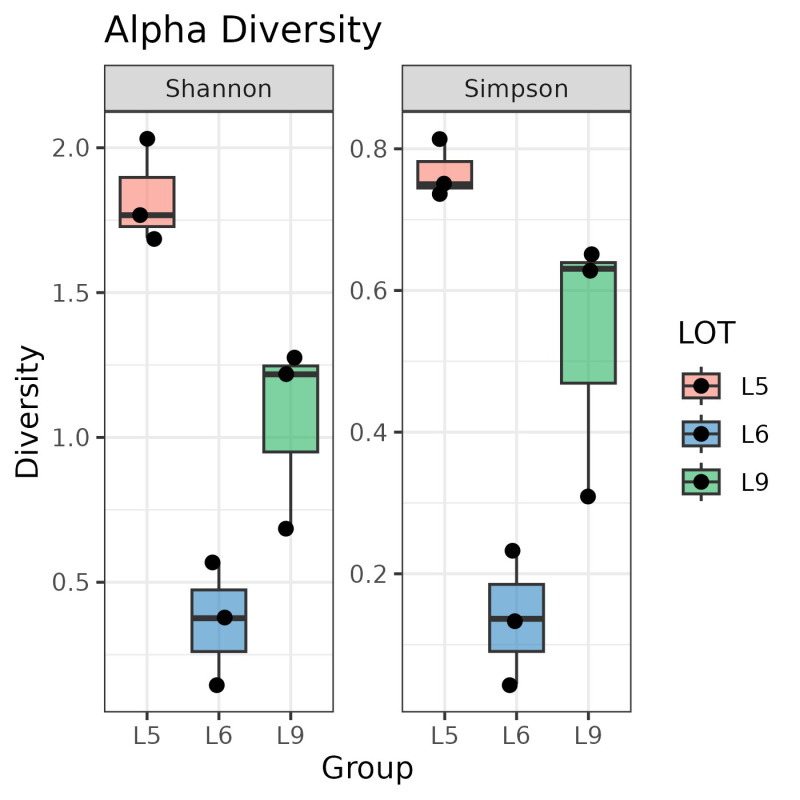
Alpha diversity indices across fermentation stages and lots. The (**left panel**) illustrates Shannon’s species richness index. The (**right panel**) displays the Simpson diversity index.

Metagenomic analyses showed that the Tuanao fermentation process was associated with phylogenetically diverse bacterial taxa. Overall, the majority of the Tuanao community was of Bacilliota and Gammaproteobacteria (specifically Enterobacterales) lineages, both of which contributed to >99% of the relative abundance in the community (Figure 3). Samples from Lot 6 were primarily occupied by members of Bacilliota, while samples from Lot 5 and Lot 9 shared a mixture of both Bacilliota and Enterobacterales, suggesting that a variety of bacterial groups, rather than specific ones, contributed to the Tuanao fermentation process. Results also showed an obvious pattern of bacterial shifts throughout the fermentation course. During the fermentation process, samples from Lot 5 demonstrated an increase in Enterobacterales and a decrease in Bacillota, while samples from Lot 9 displayed the opposite trend, with an increase in *Enterobacterales* and a decrease in Bacilliota. Meanwhile, samples from Lot 6, where Bacilliota was almost the sole component, showed a small bacterial shift that occurred among a few genera such as *Bacillus*, *Lysinibacillus*, *Nialia*, and *Caldibacillus*. In addition, the highest bacterial alpha diversity was observed in Lot 5, followed by Lot 9 and Lot 6, yet there was no consistent trend of Shannon alpha diversity between fermentation lots or days (Figure 4).

#### 3.2.2. Metabolic Components Differed by Stages of Fermentation

**Figure 5:**
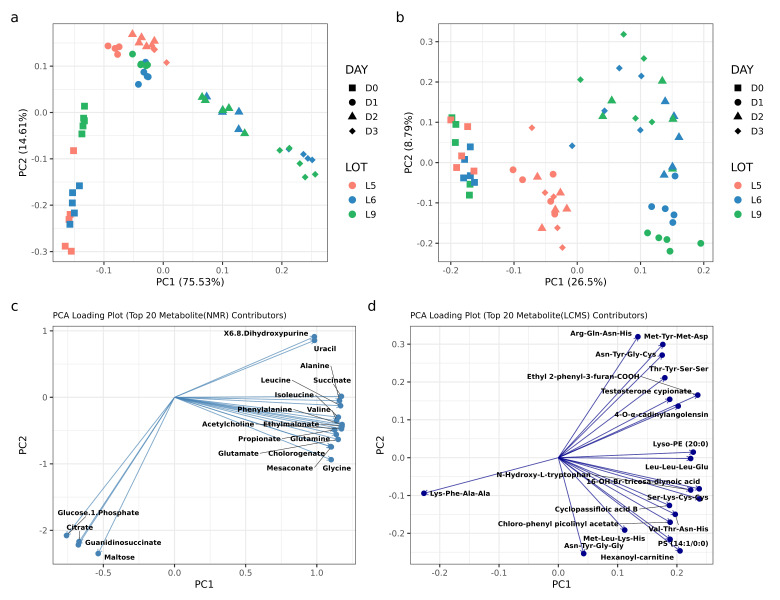
(**a**) Principal component analysis (PCA) results of metabolites from soybean samples, as detected by (**a**) NMR and (**b**) LC-MS, categorized by different fermentation days (D1, D2, D3, D6, D9) and lots. Biplots showing relationships between loading plots of (**c**) NMR-detected metabolites and their related PCA score plot and (**d**) LC–MS-detected metabolites and their related PCA score plot.

Principal component analysis showed that all fermented Tuanao lots displayed a clear shift in metabolite components and abundances between Days 0, 1, 2, and 3 of fermentation (Figure 5a,b). Lots 6 and 9 exhibited a similar degree of compositional variations during the fermentation period, while componental changes in Lot 5 tended to be smaller, especially from Days 1, 2, and 3. Moreover, an NMR PCA biplot showed that different stages of the Tuanao fermentation process were associated with distinct metabolites (Figure 5c), with citrate, maltose, guanidinosuccinate, and glucose 1-phosphate being related to Day 0 of all Lots. A group of metabolites, including glycine, mesaconate, glutamine, glutamate, propionate, valine, phenylalanine, acetylcholine, and chlorogenate, was observed to be related to Day 2 of Lots 6 and 9. Additionally, uracil and 6,8-dihydroxypurine tended to be associated with Day 1 of all lots, as well as Days 2 and 3 of Lot 5. Likewise, LC-MS PCA biplot data also confirmed the differences in metabolic contents between each stage of fermentation (Figure 5d), especially from Day 1 onwards. Examples of metabolites specially associated with Day 1 of Lot 6 and 9 included hexanoyl-carnitine, phosphatidylserine, and Chlorophenyl picolinylacetate. On the other hand, Days 2 and 3 of Lots 6 and 9 were particularly influenced by ethyl 2-phenyl-3-furan-COOH, testosterone cypionate, 4-O-α-cadinylangolnsin, and certain sets of oligopeptides.

Consistently, results from NMR profiling revealed that the metabolite abundance of each Tuanao lot differed between days (Figure 2a). A sharp increase in the acetate proportion was observed after 24 h of fermentation, followed by a gradual decrease throughout the rest of the course. Notably, the proportions of guanidinosuccinate, glucose 1-phosphate, and citrate were the majority at Day 0, yet their abundances dramatically decreased after 24 h of fermentation and remained relatively low until the end of Day 3. On the contrary, the fractions of succinate, leucine, alanine, acetylcholine, valine, and glutamine were relatively small at Day 0 before increasing after 24 h and remained so throughout the process. Correspondingly, analysis by LC-MS demonstrated changing trends in other metabolites from the Tuanao fermentation process (Figure 2b). For example, Succinylsulfathiazole, which was among the primary metabolic components as shown by LC-MS, was shown to have a gradually decreasing abundance, while a small shift in the sulfide B 823-08 relative abundances was observed in Lots 6 and 9. In contrast, the fractions of certain metabolic components, such as 12-Deoxyogalonic acid, Italipyrone, Thiophanate, including certain oligopeptides, remained constant throughout the fermentation period. Taken together, our results showed that while the overall metabolic compositions of fermented Tuanao were somewhat similar among lots (or the sources of soybeans), the metabolite abundances transformed over the time of the fermentation period.

#### 3.2.3. Metabolic Contents Significantly Correlated with Bacterial Groupings

**Figure 6:**
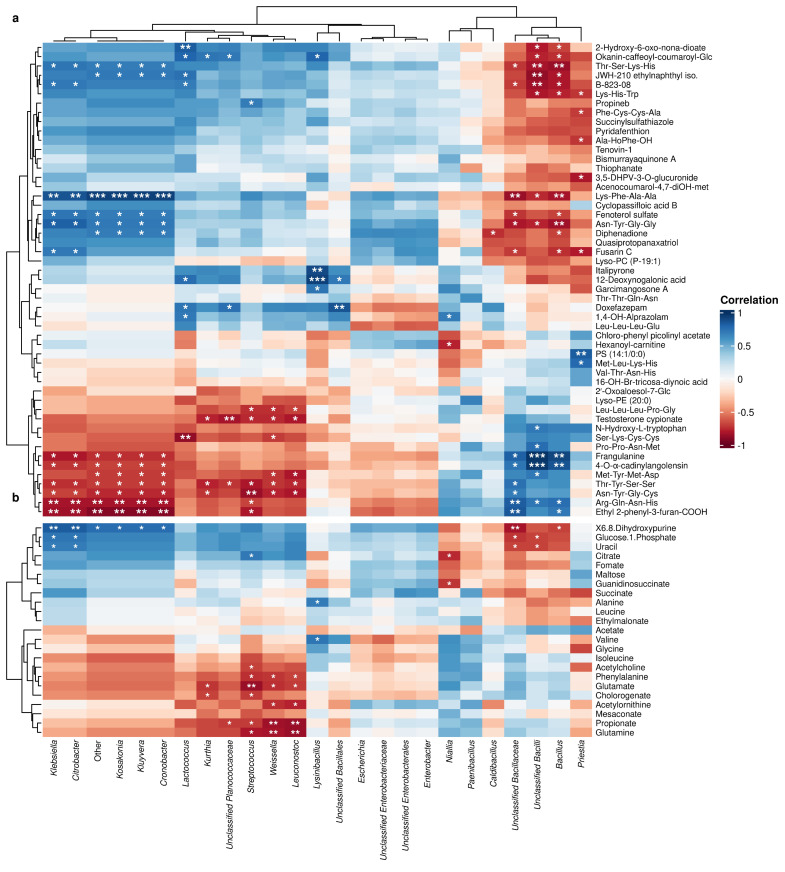
Spearman’s correlation analysis between bacterial genera and metabolites as detected by (**a**) LC-MS and (**b**) NMR. Statistically significant correlations (*p* < 0.05, *p* < 0.01, and <0.001) are marked with asterisks (*, **, and ***, respectively).

Spearman’s correlation analysis showed several strong positive and negative correlations between multiple metabolites, as detected by LC-MS, and certain groups of bacteria (Figure 6). For example, a group of *Klebsiella*, *Citrobacter*, *Kosakonia*, *Kluyvera*, and *Cronobacter* altogether demonstrated significantly negative correlations with Ethyl-2-phenyl-3-furan-COOH, Franfulanine, and certain oligopeptides, while exhibiting significantly positive correlations with Fenoterol sulfate, sulfide B-823-08, and other sets of oligopeptides. On the contrary, another bacterial group consisting of *Bacillus* and its other unclassified relatives instead displayed opposite trends, with strong positive correlations with Ethyl-2-phenyl-3-furan-COOH, Franfulanine, and such groups of oligopeptides and significantly negative correlations with Fenoterol sulfate, sulfide B-823-08, and such sets of oligopeptides. On the other hand, Spearman’s correlation analysis between bacteria and metabolites, as analyzed by NMR, showed fewer significant correlations compared to those identified by LC-MS. Of these, a group of *Escherichia, Enterobacter,* and other unclassified *Enterobacter* relatives showed a few to no significant correlations from both LC-MS and NMR analyses. However, these correlations of metabolites derived from NMR analysis still had bacterial combinations similar to those from LC-MS analysis. Moreover, the strong positive, negative, or non-existing trends were also somewhat comparable between each bacterial grouping of each analyzing technique, suggesting that these specific combinations of bacteria might altogether contribute to the abundances of certain metabolites and oligopeptides.

**Figure 7:**
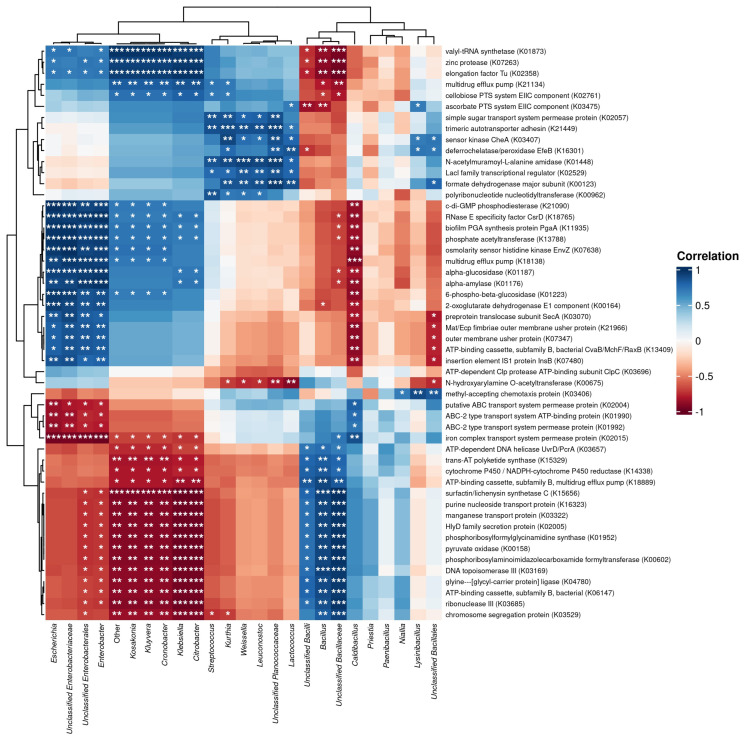
Spearman’s correlation analysis between bacterial genera and genes. Statistically significant correlations (*p* < 0.05, *p* < 0.01, and *p* <0.001) are marked with asterisks (*, **, and ***, respectively).

In addition, Spearman’s correlations also revealed the associations of certain groupings of bacteria and their genes, including certain groupings of genes and microbes (Figure 7). *Bacillus* species, along with closely related genera such as *Priestia*, *Paenibacillus*, and *Niallia*, exhibited robust positive correlations with genes involved in nutrient uptake, notably the oligopeptide (*Opp* system) and dipeptide (*Dpp* system) transporters, including *oppA*, *oppB*, *oppC*, *oppD*, and *oppF*, as well as *dppA* through *dppF* (K01990–K01995, K02039–K02043). These transport systems are crucial for internalizing soy-derived peptides, aligning with *Bacillus*’s recognized proteolytic capabilities in soybean fermentations, subsequently releasing free amino acids that significantly contribute to Tuanao’s sensory attributes, particularly umami and bitterness [2,15].

Additionally, the *Bacillus* group showed strong positive correlations with genes involved in carbohydrate metabolism, particularly fructose metabolism through the fructose-specific phosphotransferase system (PTS) encoded by *fruA* and fructokinase *fruK* (K02777, K00869). This indicates that fructose, potentially released from the hydrolysis of soybean carbohydrates, is a vital energy source supporting *Bacillus*’s dominant fermentation activity. Enzymes like alpha-glucosidase (*glcU*, K01622) and beta-glucosidase (*bglA*, K01193), important for hydrolyzing complex soy carbohydrates, were also positively correlated, reflecting *Bacillus*’s metabolic versatility. Significant correlations were also observed between *Bacillus* and fermentative pathway genes, such as L-lactate dehydrogenase (*ldh*, K00003) and alcohol dehydrogenase (*adh*, K00016). This points to *Bacillus*’s active engagement in fermentative metabolism, which likely contributes to Tuanao’s diverse metabolite profile, beyond ammonia-driven alkalization [35]. The nitrogen metabolism and amino acid biosynthesis capabilities of *Bacillus* were underscored by strong positive correlations with glutamate synthase (*gltB*, K00261) and arginine biosynthesis (*argJ*, K00623), essential in assimilating ammonia and synthesizing amino acids critical for microbial growth and flavor enhancement [36,37]. Additionally, genes involved in vitamin biosynthesis, specifically riboflavin (*ribD*, K00922) and folate (*folK*, K00790), showed positive correlations with *Bacillus*, suggesting that microbial vitamin synthesis significantly enhances the nutritional profile of Tuanao [38]. Interestingly, contrasting correlation patterns emerged with lactic acid bacteria (LAB), such as *Weissella* and *Leuconostoc*. These LAB displayed strong negative correlations with *Bacillus*-favored genes like fructose-specific PTS, yet positive correlations with ldh, indicating their specialized roles in lactic acid production and early-stage acidification of the fermentation environment [39].

Lastly, *Enterobacteriaceae* members, notably *Klebsiella* and *Enterobacter*, exhibited positive correlations with fermentative genes like *adh* and *ldh*, suggesting their involvement primarily during early fermentation stages. *Kosakonia* uniquely correlated positively with *araH*, a sugar transport gene, highlighting niche specialization by exploiting specific carbohydrates like arabinose, differentiating their ecological role from dominant *Bacillus* populations [35].

## 4. Discussion

### 4.1. Unveiling the Complexity of Tuanao Fermentation Through an Integrated Omics Lens

This study employed an integrated metagenomics and metabolomics approach to dissect microbial and biochemical transformations in traditional Tuanao fermentation, providing unprecedented detail on microbial succession, genetic potential, and the evolving metabolite landscape. This dual omics strategy was synergistic: metagenomics identified microbial species and their functional genes, while metabolomics captured the biochemical output. Correlation analyses bridged these layers, exemplified by linking *Bacillus* dominance to peptide transport genes and the subsequent appearance of oligopeptides and free amino acids. This progression from microbial presence to genetic capability and biochemical impact offers a robust, mechanistic understanding unachievable by single omics methods, highlighting the power of systems biology in studying traditional food fermentations and understanding their functions, flavors, and qualities [40].

### 4.2. The Microbial Ecosystem of Tuanao: A Dynamic Succession Orchestrated Primarily by Bacillota

Metagenomic analysis identifies *Bacillota*, particularly *Bacillus* species, as the principal architects of Tuanao fermentation, with their abundance increasing significantly with progression, consistent with the literature on similar fermented soybeans. Early fermentation shows a transient presence of *Gammaproteobacteria* (*Enterobacterales*), which, as facultative anaerobes, likely create anaerobic conditions favoring later microbial growth [41]. These are outcompeted by *Bacillus* due to environmental shifts like pH decrease, oxygen depletion, and potential antimicrobial production [42]. This succession to *Bacillus* dominance is crucial for Tuanao’s sensory properties and microbiological safety.

*Bacillus*’s functional contributions are inferred from its genetic potential. Strong positive correlations link *Bacillus* and its related genera (*Priestia*, *Paenibacillus*, *Niallia*) with genes for oligopeptide (Opp) and dipeptide (Dpp) transport systems, crucial for soy protein degradation and peptide uptake [43]. This contributes to the accumulation of free amino acids and peptides. *Bacillus*’s carbohydrate metabolism proficiency is shown by correlations with the fructose-specific PTS (*fruA*, *fruK*) and hydrolytic enzymes like alpha-glucosidase (*glcU*) and beta-glucosidase (*bglA*), used for fructose transport and complex carbohydrate breakdown [44]. This indicates active utilization of soy carbohydrates for energy and dominance.

*Bacillus*’s association with L-lactate dehydrogenase (*ldh*) and alcohol dehydrogenase (*adh*) genes suggests broader metabolic contributions beyond typical ammonia production in alkaline fermentations, including organic acid production [45]. Its capacity for nitrogen metabolism and amino acid biosynthesis is shown by correlations with glutamate synthase (*gltB*) and arginine biosynthesis (*argJ*) genes, vital for ammonia assimilation and synthesizing new amino acids, enhancing nutritional quality and flavor. Significantly, *Bacillus* correlates with riboflavin (*ribD*) and folate (*folK*) biosynthesis genes, suggesting de novo synthesis of these B vitamins, enriching Tuanao’s nutritional value. *Bacillus*’s dominance is thus functional, driven by a versatile genetic arsenal for nutrient acquisition, energy generation, and biosynthesis, reshaping the soybean matrix. This toolkit offers targets for process optimization.

Other microbes contribute to fermentation dynamics. Lactic acid bacteria (LAB) like *Weissella* and *Leuconostoc* showed negative correlations with *Bacillus*-favored genes (e.g., fructose PTS) but positive correlations with *ldh*, indicating specialized lactic acid production, early acidification, and flavor development, potentially modulating *Bacillus* activity. Early-stage *Enterobacteriaceae* (*Klebsiella*, *Enterobacter*) also correlated with *adh* and *ldh*, contributing initial fermentation byproducts. *Kosakonia*’s unique positive correlation with sugar transporter *araH* suggests arabinose utilization, indicating niche specialization. This initial *Enterobacterales* bloom may precondition the environment by consuming sugars and depleting oxygen [41], facilitating *Bacillus*’s subsequent dominance and influencing final Tuanao characteristics.

Variability in microbial profiles and succession was evident across fermentation lots—for example, Lot 5 versus Lot 9—without a consistent trend in Shannon α-diversity (Figure 3). Although Bacillus generally emerged as the dominant genus, the trajectory and supporting taxa differed from batch to batch. The pronounced lot-to-lot variability observed in both microbial succession and metabolite profiles is a central finding of this study, directly reflecting the artisanal nature of traditional Tuanao production. Unlike industrial processes that rely on defined starter cultures, spontaneous fermentation is inherently stochastic, influenced by the indigenous microbiome of the raw soybeans and the immediate environment. This variability can be viewed as a double-edged sword: it is the likely source of the unique and complex sensory attributes that distinguish batches and are prized by consumers, yet it also presents a significant hurdle for achieving product consistency for industrial scale-up. By characterizing this variability at a molecular level, our findings provide a crucial baseline for future efforts to standardize Tuanao production, for instance, through the development of starter cultures that can ensure safety and consistency while preserving the core sensory characteristics of this traditional food.

Using different soybean cultivars can further modulate texture and microbial succession, as demonstrated when seeds were inoculated with B. natto “Itobiki” [46]. Although natto harbors a diverse microbiota, *Bacillus subtilis* remains the pivotal driver of fermentation; the species itself comprises hundreds of intraspecific variants that shape community composition [47]. To minimize raw material variability, we standardized the substrate by sourcing all beans from a single nationwide distributor. Nevertheless, even with this control, our shotgun metagenomic data revealed discernible shifts in community structure between batches, indicating that factors beyond cultivar, such as micro-niches within beans or stochastic colonization events, still influence the fermentative microbiome.

### 4.3. The Evolving Metabolome: Signatures of Microbial Biotransformation and Nutritional Enhancement

Fermentation induced profound, dynamic shifts in the Tuanao metabolome, mapped by NMR and LC-MS. An early key event was a sharp NMR-detected acetate increase around 24 h, indicating active carbohydrate fermentation by early consortia, possibly *Enterobacterales* and emerging *Bacillus*, via pathways like phosphotransacetylase/acetate kinase or succinyl-CoA: acetate CoA-transferase [48]. Later, hallmark transformations included significant accumulation of free amino acids (glutamine, leucine, alanine, valine, phenylalanine by NMR and diverse oligopeptides (LC-MS, Days 2–3). This results from extensive proteolysis of soy proteins by *Bacillus* enzymes. Conversely, initial substrates like citrate and glucose-1-phosphate decreased, being consumed by microbes. This sequential appearance of metabolite classes reflects microbial succession and phased enzymatic activities, with early carbohydrate fermentation paving the way for later *Bacillus*-driven proteolysis. The temporal metabolic profile snapshots predominant microbial functions.

Complementary NMR and LC-MS offered a comprehensive metabolome characterization. NMR quantified abundant primary metabolites (amino acids, organic acids, sugars), tracking major shifts. High-resolution UHPLC-ESI-QTOF-MS/MS detected a broader array of compounds, including oligopeptides, acylcarnitines, fatty acids, and steroids, often at lower concentrations. This dual strategy captured large-scale transformations and subtle chemical signatures. LC-MS revealed more significant microbe–metabolite correlations than NMR, likely due to its higher sensitivity to specific secondary metabolites and diverse peptide products. While NMR tracks central metabolism, LC-MS excels at detecting diverse, lower-abundance specialized metabolites. Thus, a multi-platform approach is invaluable for a complete understanding of microbial impact on food metabolomes.

Spearman’s correlations provided direct evidence of microbe–metabolite linkages. *Bacillus* species showed strong positive correlations with certain oligopeptides, Ethyl-2-phenyl-3-furan-COOH, and Franfulanine and negative correlations with Fenoterol sulfate and sulfide B-823-08, highlighting its central role in Tuanao’s chemical profile. Conversely, *Klebsiella*, *Citrobacter*, *Kosakonia*, *Kluyvera*, and *Cronobacter* showed opposite trends, suggesting distinct contributions and potential interactions. The diversity of LC-MS-detected oligopeptides correlated with *Bacillus* abundance is noteworthy, suggesting a rich repertoire of potentially bioactive peptides beyond increased free amino acids. *Bacillus* proteases generate diverse peptide sequences from soy proteins, many of which could be short, bioactive peptides with ACE-inhibitory, antioxidant, or immunomodulatory properties [49]. This implies *Bacillus* produces specific peptide patterns crucial for Tuanao’s functionality. Future research should identify these oligopeptides to understand their health and flavor contributions.

### 4.4. Unveiling the Health-Promoting Potential of Tuanao: A Mechanistic Perspective Based on Integrated Omics

Our integrated profiles provide a molecular basis for exploring Tuanao’s potential as a functional food, offering mechanistic hypotheses for its purported health benefits. Dominant *Bacillus* species, many of which are GRAS or probiotic, may deliver beneficial microbes/spores to the gut, modulating microbiota and enhancing barrier function [50]. Tuanao is also rich in postbiotic metabolites. Early-peaking acetate, a key SCFA, benefits gut health by lowering pH and nourishing colonocytes [51]. Diverse peptides and synthesized vitamins further contribute to its postbiotic effects. Bioactive peptides from soy protein hydrolysis are major health contributors. Increased oligopeptides, correlated with *Bacillus* and its peptide-processing genes (*Opp*/*Dpp* systems), indicate Tuanao is a rich source. These peptides, common in fermented soy, can have ACE-inhibitory, antioxidant, and immunomodulatory effects [49]. This study links *Bacillus* to these peptide families.

Fermentation enhances Tuanao’s nutritional value, especially amino acid and vitamin content. Increased free amino acids (glutamine, leucine, alanine, valine, phenylalanine) improve soy protein digestibility and nutritional profile. Glutamine supports gut integrity and immune function. Essential amino acids like phenylalanine improve protein quality. Strong correlation between *Bacillus* and riboflavin (*ribD*) and folate (*folK*) synthesis genes evidences de novo B-vitamin production [38], mechanistically linking microbial genetics to nutritional enhancement. Dominant *Bacillus* species, likely related to *B. subtilis*, imply production of other bioactive compounds. These include nattokinase, a fibrinolytic enzyme beneficial for cardiovascular health, and poly-γ-glutamic acid (PGA), a viscous biopolymer with prebiotic and immunomodulatory potential [52]. *Bacillus* dominance reinforces Tuanao as a source of these compounds.

Conversion of soy isoflavone glycosides to more bioavailable aglycones (daidzein, genistein) by microbial β-glucosidases is another health benefit. The positive correlation between *Bacillus* and a β-glucosidase gene (*bglA*, K01193) mechanistically links this biotransformation in Tuanao. Aglycones have superior antioxidant activity and phytoestrogenic effects, supporting bone health, alleviating menopausal symptoms, and potentially offering anti-cancer properties [53].

This research provides mechanistic evidence from integrated omics data, moving beyond mere cataloging. For instance, oligopeptide production is correlated with *Bacillus* species possessing peptide-processing genes (*Opp*/*Dpp* systems). Enhanced vitamin content is linked to specific biosynthesis genes in dominant *Bacillus*, and increased isoflavone bioavailability to their β-glucosidase activity. Tracing traits from microbe to gene to metabolite strengthens claims about Tuanao’s functional properties, grounding them in study-specific data. Tuanao’s diverse beneficial compounds (probiotic *Bacillus*, acetate, bioactive peptides, free amino acids, B vitamins, likely nattokinase/PGA, isoflavone aglycones) suggest the potential for synergistic health benefits. Multiple components acting on various pathways may create a “food matrix effect,” making whole Tuanao consumption more beneficial than isolated components, promoting it as a valuable functional food.

To synthesize these direct links, Table 1 summarizes key microbial contributions to Tuanao’s functional profile as elucidated by this study.

### 4.5. Tuanao in the Context of Fermented Soy Foods and Scientific Advancements

Tuanao’s microbial and metabolite profiles allow comparisons with other asian fermented soybeans (natto, cheonggukjang, kinema), where *Bacillus* species, especially *B. subtilis*, dominate core transformations like proteolysis and isoflavone bioconversion. This study confirms *Bacillus* as Tuanao’s keystone genus. However, detailed omics data reveal unique features: specific oligopeptide families correlated with *Bacillus* and distinct roles of minor groups like *Kosakonia* (arabinose utilization via *araH*) may create a unique fingerprint. Indigenous Tuanao strains and their enzyme production (nattokinase, PGA) might differ from those in other products, causing variations [16]. While sharing core features, Tuanao’s multi-omics signature (unique microbe–metabolite correlations, minor population influences) suggests each traditional product has a subtly unique ecosystem. These distinctions, from variations in raw materials, microflora, and practices, yield distinct sensory and functional nuances [54]. This study provides tools for dissecting these distinctions, which is crucial for appreciating diversity and preserving unique qualities.

This research advances the methodology for understanding complex food fermentations. Integrated shotgun metagenomics, dual-platform metabolomics, and bioinformatics provide a holistic, mechanistic understanding of Tuanao fermentation, surpassing single omics or culture-based methods. It moves from identifying microbes to elucidating their genetic potential and actual biochemical transformations and linking genetic attributes to activities. This comprehensive omics pipeline, successfully applied to Tuanao, serves as a blueprint for studying other traditional fermented foods globally. This approach can help preserve microbial diversity, identify novel probiotics or bioactives, and optimize traditional processes for safety, consistency, and functionality.

### 4.6. Limitations and Future Research Trajectories

While metagenomics revealed the genetic potential of Tuanao, it did not exclusively unveil gene expression levels and active pathways, which could be confirmed through metatranscriptomics. Furthermore, a key limitation of our metabolomics analysis is that the LC-MS annotations are tentative. While this untargeted approach provided a broad overview of the chemical landscape, definitive structural identification and absolute quantification would require further validation using MS/MS fragmentation and comparison to pure standards. Consequently, the correlations observed between microbes and these putative metabolites should be interpreted as strong hypotheses that require targeted follow-up studies. Inferred health properties require direct validation via in vivo or human clinical trials.

Future research should include isolating and characterizing key *Bacillus* and other microbes (e.g., *Kosakonia*) and investigating their enzymatic activities and bioactive compound production in vitro. Exploring the impacts of varied raw materials and fermentation parameters on microbial communities, gene expression, metabolites, and sensory properties is also vital. Metatranscriptomics integrated with metabolomics can bridge genetic potential and activity, pinpointing active pathways and responsible organisms. Sensory analysis correlated with omics data can identify flavor/aroma drivers. Essential in vivo studies are needed to translate inferred health benefits into evidence-based claims, assessing Tuanao’s effects on gut microbiota, immune responses, blood pressure, or glucose metabolism. Investigating roles and interactions of less dominant microbes (LAB, other *Bacillota*) could reveal their contributions. Prioritizing research linking omics findings to tangible outcomes (starter cultures, validated health benefits) is crucial for translating this knowledge into practical applications and scientific advancements.

## 5. Conclusions

The integrated omics investigation provides a deep characterization of Tuanao’s microbial ecology and biochemical transformations, demonstrating that Tuanao fermentation is a dynamic, *Bacillus*-driven process that transforms soybeans into a nutritionally enhanced food rich in potentially health-promoting compounds. By elucidating microbe–gene–metabolite networks, this study offers mechanistic insights into how beneficial attributes are generated, which is significant for preserving and promoting traditional fermented foods like Tuanao through scientific validation, providing a basis for developing novel functional foods or starter cultures to optimize bioactive compound production and ensure consistency/safety, and informing dietary guidelines with evidence of nutritional enrichment and bioactive molecule generation, thereby encouraging Tuanao’s inclusion for improved nutrition and disease prevention. Ultimately, this study positions Tuanao as a complex, microbially engineered functional-food system, where the interplay of microbial communities, genetics, and metabolic transformations yields a product with numerous health benefits, a perspective that can elevate Tuanao’s status and highlights the value of integrating traditional knowledge with advanced science to enhance food security and nutrition and harness microbial power.

## Figures and Tables

**Table 1:** Summary of key microbial contributions to the functional profile of Tuanao.

Microbial Group/Key Genus	Key Associated Genes/Pathways from This Study (KEGG Orthology ID)	Key Correlated/Produced Metabolites from This Study	Inferred Functional Role and Health Implication
*Bacillus* spp. (and related *Bacillota*)	Oligopeptide transport system (*Opp*; K01990–K01995), Dipeptide transport system (*Dpp*; K02039–K02043)	Increased free amino acids (glutamine, leucine, alanine, valine, phenylalanine), specific oligopeptide groups	Soy protein hydrolysis and generation of bioactive peptides (potential for ACE inhibition, antioxidant activity); enhanced amino acid availability and improved nutritional quality; contribution to umami and other flavor characteristics.
*Bacillus* spp.	Fructose-specific PTS system (*fruA*; K02777), Fructokinase (*fruK*; K00869), Alpha-glucosidase (*glcU*; K01622)	Consumption of soy carbohydrates, production of acetate (early phase)	Efficient utilization of soy carbohydrates for energy to drive fermentation; contribution to organic acid profile and initial pH modulation.
*Bacillus* spp.	β-glucosidase (*bglA*; K01193)	Potential increase in isoflavone aglycones (e.g., daidzein, genistein)	Conversion of isoflavone glycosides to more bioavailable and bioactive aglycone forms; enhanced antioxidant capacity and potential for phytoestrogenic and other health benefits.
*Bacillus* spp.	Riboflavin biosynthesis protein (*ribD*; K00922), Dihydroneopterin aldolase (*folK*; K00790)	Potential de novo synthesis of riboflavin (Vitamin B2) and folate (Vitamin B9)	Microbial enrichment of Tuanao with essential B vitamins; enhanced overall nutritional value of the fermented product.
*Bacillus* spp.	Glutamate synthase (*gltB*; K00261), Arginine biosynthesis bifunctional protein (*argJ*; K00623)	Increased free amino acids (e.g., glutamine)	Assimilation of nitrogen and de novo synthesis of amino acids; contribution to flavor development and enhanced protein quality.
*Enterobacterales* (early stage), *Bacillus* spp.	L-lactate dehydrogenase (*ldh*; K00003), Alcohol dehydrogenase (*adh*; K00016)	Acetate, potentially other organic acids and alcohols	Contribution to early-stage fermentation byproducts, flavor development, and environmental modification (e.g., pH, redox potential) that influences microbial succession.
*Weissella*, *Leuconostoc* (LAB)	L-lactate dehydrogenase (*ldh*; K00003)	Lactic acid (inferred)	Potential for lactic acid production, contributing to acidification, flavor complexity, and inhibition of spoilage/pathogenic microbes, particularly in specific niches or early stages.
*Kosakonia*	Sugar transport system permease protein (*araH*)	Utilization of specific sugars (e.g., arabinose, inferred)	Niche specialization by utilizing specific carbohydrate substrates not readily used by other dominant microbes; contribution to overall substrate degradation and microbial diversity.

## Data Availability

Raw metagenomic sequences can be accessed through NCBI under BioProject PRJNA1268715.

## Supplementary Materials

The following supporting information can be downloaded at https://www.mdpi.com/article/10.3390/foods14173070/s1: Table S1: Metabolites identified by NMR spectroscopy in Tuanao fermentation samples; Table S2: Metabolites detected by UHPLC-ESI-QTOF-MS/MS in Tuanao fermentation samples. Figure S1: Chromatogram from the LC-MS analysis of a representative *Tuanao* sample of each day from all fermentation batch.
