# Supplementary material for: Insights into Microbial and Metabolite Profiles in Traditional Northern Thai Fermented Soybean (Tuanao) Fermentation Through Metagenomics and Metabolomics
Source: Foods. 2025 Aug 30;14(17):3070. doi: 10.3390/foods14173070 (PMC12428203; doi:10.3390/foods14173070)
Supplement: Supplementary file 1 [file foods-14-03070-s001.zip › Table S1.pdf]

Insight into Microbial and Metabolite Profiles in Traditional Northern Thai Fermented Soy beans (Tuanao) Fermentation Through Metagenomics and Metabolomics

Table S1 Metabolite identification

NO	Metabolite	Chemical Shift	Driver peak (ppm)
1	Isoleucine	0.90555 (t), 1.02 (d), 1.276 (m), 1.46 (m), 1.981 (m), 3.683 (d)	0.90555
2	Leucine	0.9429 (t), 1.708 (m), 3.729 (t)	0.9429
3	Valine	0.99336 (d), 1.022 (d), 2.25 (dh), 3.621 (d)	0.99336
4	Unknown 1	1.1323 (m), 1.329 (d)	1.1323
5	Ethanol	1.171 (t) 3.643 (q)	1.171
6	Alanine	1.4859 (d), 3.74 (qt)	1.4859
7	Acetate	1.932 (s)	1.932
8	Glutamine	2.052 (m), 2.458 (m), 3.763 (t)	2.052
9	Acetylcholine	2.1493 (s), 3.19 (s), 3.739 (m)	2.1493
10	Propionate	1.041 (t), 2.181 (q)	2.181
11	Glutamate	2.105 (m), 2.3179 (dt),3.76 (t)	2.3179
12	Succinate	2.4057 (s)	2.4057
13	Citrate	2.4962 (d), 2.652 (d)	2.4962
14	Ethylmalonate	1.67 (m), 3.0066 (t), 0.846 (t)	3.0066
15	Acetylornithine	1.708 (m), 1.903 (m), 2.019 (s), 3.0066 (t), 4.1218 (m), 7.897 (m)	4.1218
16	Methanol	3.3595 (s)	3.3595
17	Maltose	3.4739 (t), 3.588 (m), 3.696 (m), 3.823 (m), 3.924 (m), 5.412 (d)	3.4739
18	Glycine	3.5795 (s)	3.5795
19	Guanidinosuccinate	2.5 (dd), 2.828 (dd), 4.2295 (dd)	4.2295
20	Glucose-1-Phosphate	3.47 (t), 3.56 (m), 3.72 (m), 3.84 (m), 3.9 (m), 5.4117 (dd)	5.4117
21	Uracil	5.8121 (d), 7.59 (d)	5.8121
22	Cholorogenate	1.98 (m), 2.11 (d), 2.14 (m), 3.86 (dd), 4.302 (d), 5.27 (td), 6.411 (s), 6.45 (s), 6.93 (d), 7.19 (dd), 7.29 (s), 7.596 (d)	6.411
23	Mesaconate	1.932 (s), 6.4516 (s)	6.4516
24	Phenylalanine	3.125 (dd), 3.295 (dd), 4.13 (dd), 7.3398 (m)	7.3398
25	6,8-Dihydroxypurine	8.2553 (s)	8.2553
26	Formate	8.477 (s)	8.477

Example of representative of STOCSY plot

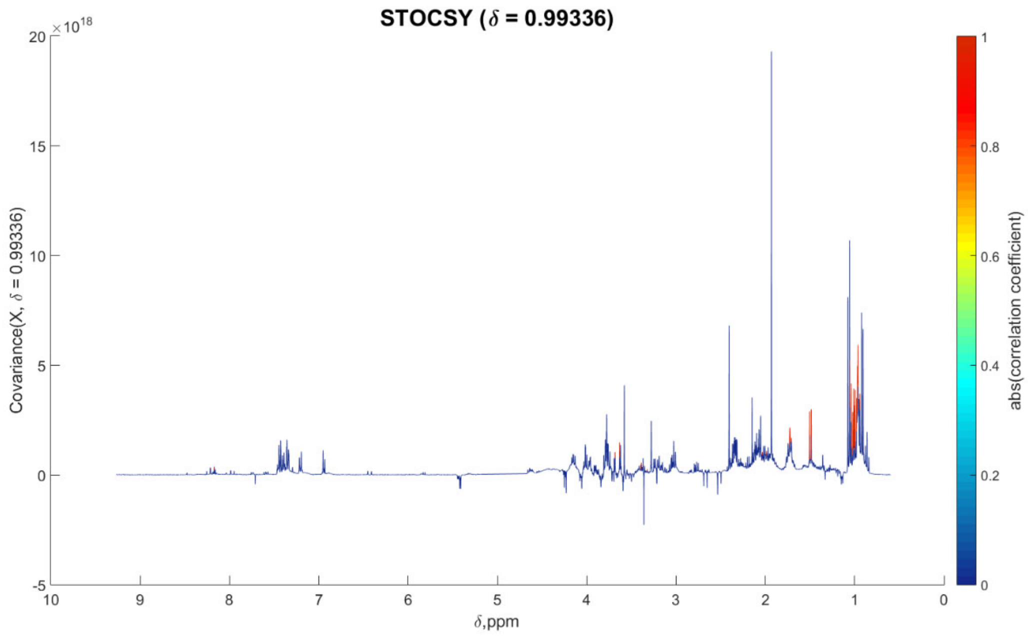
STOCSY (δ = 0.99336)
×10^18
Covariance(X, δ = 0.99336)
δ,ppm
abs(correlation coefficient)

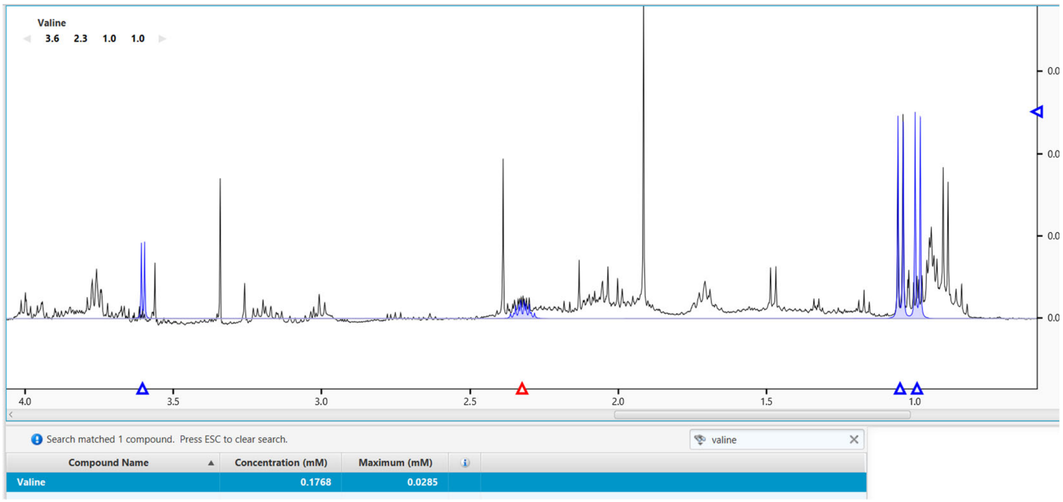
Valine
3.6 2.3 1.0 1.0
Search matched 1 compound. Press ESC to clear search.
valine
Compound Name
Concentration (mM)
Maximum (mM)
Valine 0.1768 0.0285

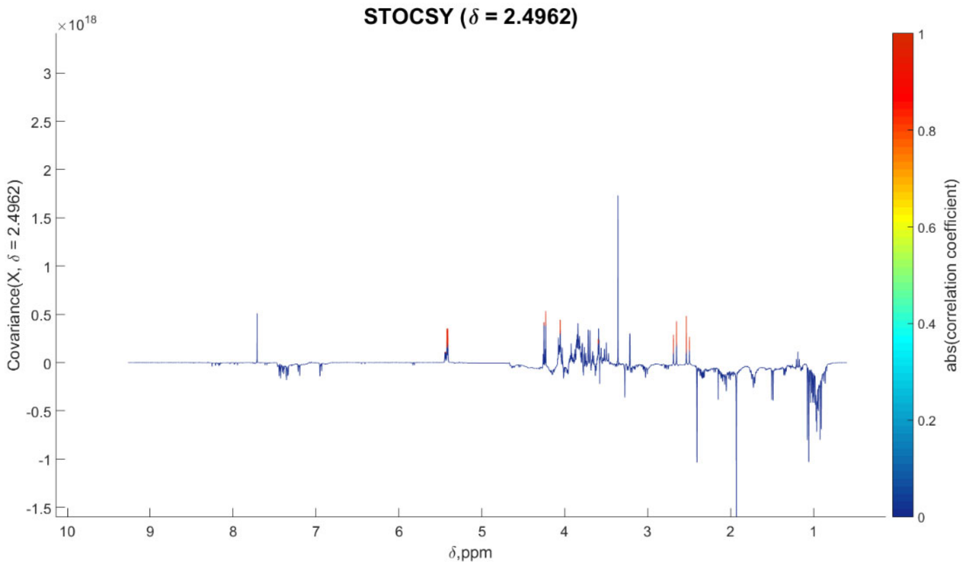
STOCSY (δ = 2.4962)
×10^18
Covariance(X, δ = 2.4962)
δ,ppm
abs(correlation coefficient)

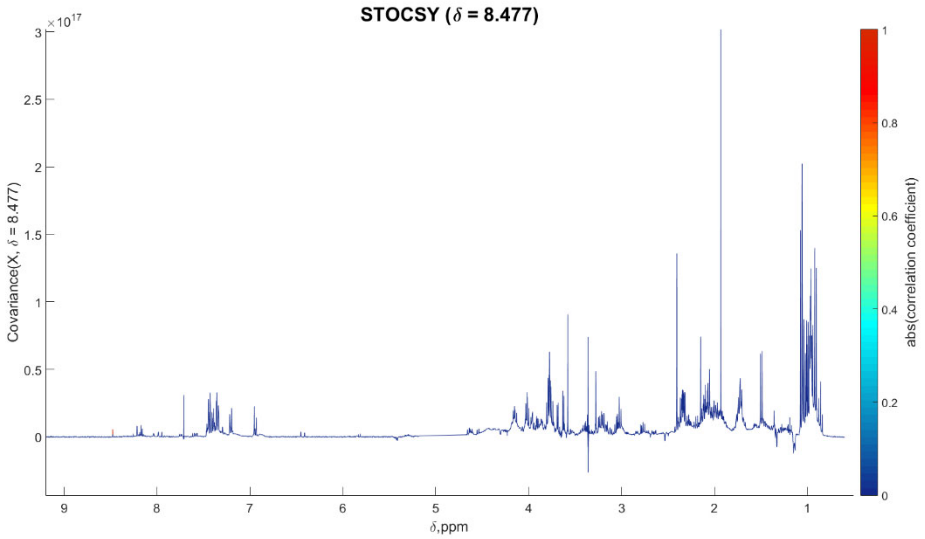
STOCSY (δ = 8.477)
$\times 10^{17}$
Covariance(X, δ = 8.477)
δ,ppm
abs(correlation coefficient)
3
2.5
2
1.5
1
0.5
0
9
8
7
6
5
4
3
2
1
0.2
0.4
0.6
0.8
