# Supplementary figures and images for: Insights into Microbial and Metabolite Profiles in Traditional Northern Thai Fermented Soybean (Tuanao) Fermentation Through Metagenomics and Metabolomics
Source: Foods. 2025 Aug 30;14(17):3070. doi: 10.3390/foods14173070 (PMC12428203; doi:10.3390/foods14173070)

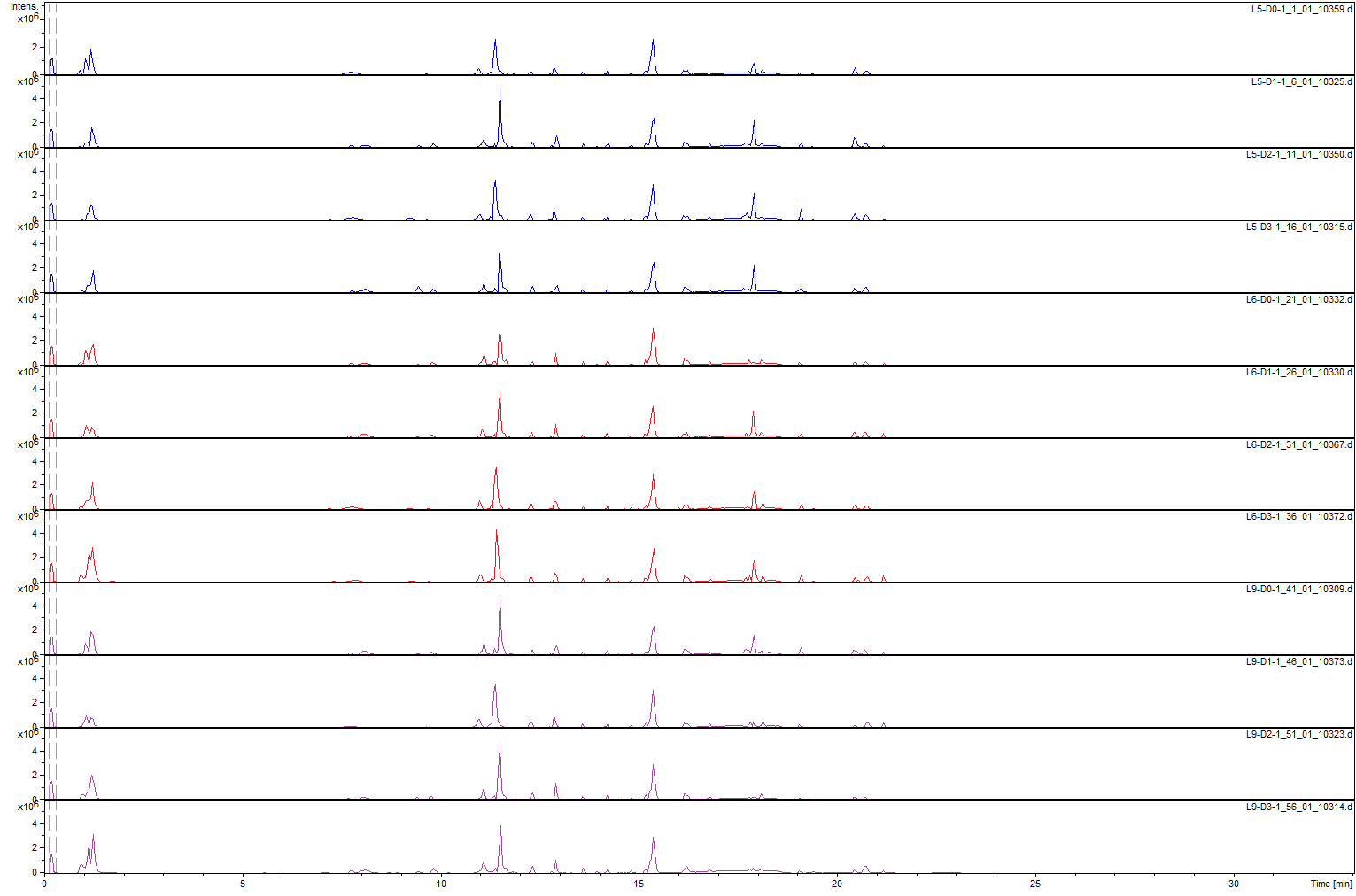

Supplement: Supplementary file 1 [file foods-14-03070-s001.zip › FigureS1.tif]
